# Discovery of Novel Integrase Inhibitors Acting outside the Active Site Through High-Throughput Screening

**DOI:** 10.3390/molecules24203675

**Published:** 2019-10-12

**Authors:** Cindy Aknin, Elena A. Smith, Christophe Marchand, Marie-Line Andreola, Yves Pommier, Mathieu Metifiot

**Affiliations:** 1 Laboratoire MFP, CNRS UMR5234, Université de Bordeaux, 146 rue Léo Saignat, 33076 Bordeaux CEDEX, France; cindy.aknin@u-bordeaux.fr (C.A.); marie-line.andreola@u-bordeaux.fr (M.-L.A.); 2 Developmental Therapeutics Branch and Laboratory of Molecular Pharmacology, CCR, NCI, NIH, 37 Convent Drive, Bethesda, MD 20892, USA; semenovel@yahoo.com (E.A.S.); marchanc@mail.nih.gov (C.M.); yves.pommier@nih.gov (Y.P.)

**Keywords:** HIV-1, INSTI resistance, DNA-binding inhibitor, high-throughput screening, drug discovery

## Abstract

Currently, an increasing number of drugs are becoming available to clinics for the treatment of HIV infection. Even if this targeted therapy is highly effective at suppressing viral replication, caregivers are facing growing therapeutic failures in patients, due to resistance with or without treatment adherence concerns. Accordingly, it is important to continue to discover small molecules that have a novel mechanism of inhibition. In this work, HIV integrase inhibitors were selected by high-throughput screening. Chemical structure comparisons enabled the identification of stilbene disulfonic acids as a potential new chemotype. Biochemical characterization of the lead compound stilbenavir (NSC34931) and a few derivatives was performed. Stilbene disulfonic acid derivatives exhibit low to sub-micromolar antiviral activity, and they inhibit integrase through DNA-binding inhibition. They probably bind to the *C*-terminal domain of integrase, in the cavity normally occupied by the noncleaved strand of the viral DNA substrate. Because of this original mode of action compared to active site strand transfer inhibitors, they do not exhibit cross-resistance to the three main resistance pathways to integrase inhibitors (G140S-Q148H, N155H, and Y143R). Further structure–activity optimization should enable the development of more active and less toxic derivatives with potential clinical relevance.

## 1. Introduction

HIV-1 integrase (IN) has become a major pharmacological target for the treatment of HIV-1 infection. IN catalyzes the insertion of viral DNA into the host chromosome, and it is critical for viral replication. Integration is carried out in two sequential steps. Immediately after reverse transcription, the newly synthesized viral DNA is cleaved by IN, releasing the terminal 3′-dinucleotide adjacent from a conserved CA dinucleotide. This reaction, called 3′-processing (3′-P), occurs in the cytoplasm of infected cells. Then, IN remains bound to the viral DNA in the preintegration complex (PIC) that migrates to the nucleus, where the second IN-mediated catalytic reaction, called strand transfer (ST), occurs (for a review, see [1]).

The approval by the US Food and Drug Administration of raltegravir (RAL, MK-0518; Isentress^®^, Merck & Co.) as the first IN inhibitor for the treatment of HIV-AIDS was a major therapeutic breakthrough (for reviews, see [2,3]). Rapidly, resistance mutations in the IN coding region have emerged (namely Y143R, Q148H, and N155H). Following these mutations, other strand transfer inhibitors were developed, but all of them share a common mechanism of action—binding in the active site of IN. Accordingly, mutations observed in patients failing therapy on RAL-based regimens confer cross-resistance to other IN strand transfer inhibitors. In the past, the development of non-nucleoside inhibitors overcame resistance to nucleoside analogs. Similar to what has been achieved in the reverse transcriptase field, an efficient way to alleviate clinical resistance to IN strand transfer inhibitors is to develop inhibitors that target IN outside of its catalytic site.

In this study, we screened the National Cancer Institute (NCI) Developmental Therapeutics Program’s plated sets from the Open Chemical Repository Collection for compounds enabled to inhibit IN strand transfer activity in vitro. Among the 130 positive hits, we rationally selected NSC34931, NSC638352, and NSC76027 for further characterization because of structural similarities. NSC34931 (stilbenavir) and other stilbene disulfonic acid derivatives exhibited antiviral activity and inhibited HIV-1 IN by targeting the viral DNA binding site on the *C*-terminal domain of the enzyme. This novel mechanism of inhibition has never been pursued to date, and specific small molecule ligands might represent a promising new therapeutic avenue.

## 2. Results

### 2.1. Screening for IN Strand Transfer Inhibitors

**Figure 1:**
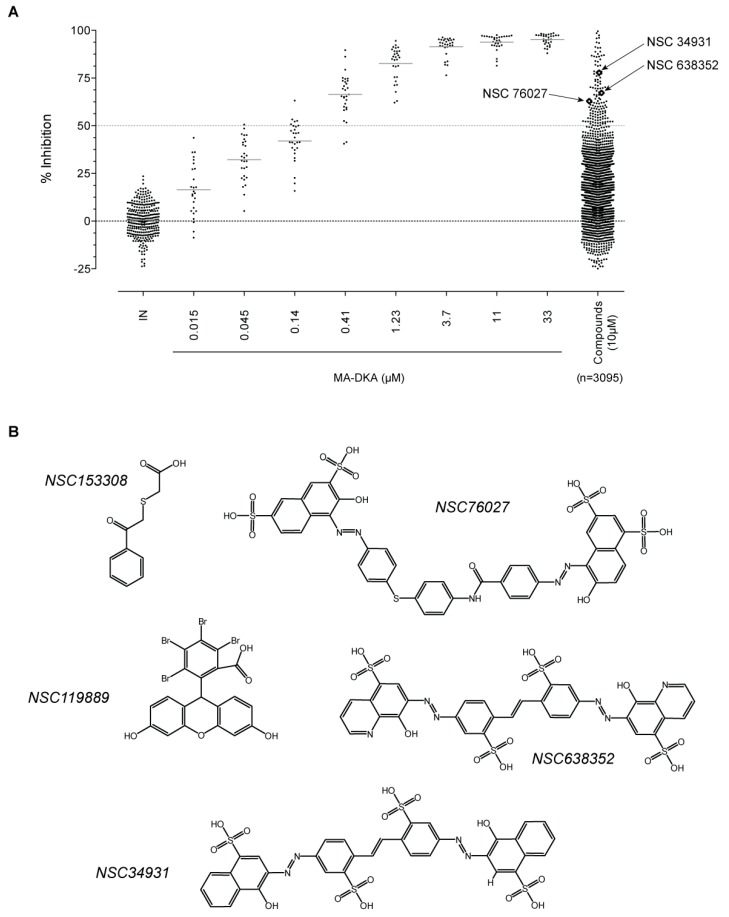
High-throughput screening of the National Cancer Institute Developmental Therapeutics Program’s 3095 small molecule plated sets from the Open Chemical Repository Collection. (**A**) Scattered plot summarizing the results of the electrochemiluminescent integrase (IN) ST screening assay. Experiments were performed in a 96-well plate format, where each plate harbored eight control points with DMSO (IN alone) and a dose response of MA-DKA (also known as 118-D-24) over eight concentrations. Altogether, the screen necessitated 39 assay plates. Open diamonds represent the three stilbene disulfonic acid derivatives identified by the screen. (**B**) Chemical structure of the two top hits and the three stilbene disulfonic acid derivatives.

To enable a high-throughput screening, we used the ST assay developed by BioVeris. This proprietary plate-based assay measured stand transfer using electrochemiluminescent technology. First, we validated the assay using HIV-1 IN in the presence of DMSO, or increasing concentrations of a known inhibitor, MA-DKA (also known as 118-D-24, Figure 1A). As expected, this diketo acid was an efficient ST inhibitor, possessing an IC_50_ of 163 nM ± 9 nM, similar to previous reports [4,5]. Next, we used MA-DKA as a control in every test plate and screened a large library of molecules. Overall, 3095 compounds were tested as part of the NCI Developmental Therapeutics Program’s small molecule plated sets from the Open Chemical Repository Collection. Interestingly, we did not find stimulating compounds. A single dose testing (10 µM) enabled the identification of 130 molecules that inhibited ST by over 50% (light dashed line, Figure 1A). The top hits were NSC153308 and NSC119889, which inhibited ST activity by 99.5% and 98.5%, respectively. As a comparison, the reference compound MA-DKA inhibited 94.8% of the IN ST activity at 10 µM (data obtained from linear regression of the dose response presented in Figure 1A). NSC153308 is a 2-phenacylsulfanylacetic acid that resembles the diketo acid chemotype. On the other side, NSC119889 is a xanthene derivative similar to V-165 [6]. Hence, both chemotypes have previously been reported, which validated the capacity of the screen to identify IN specific inhibitors (Figure 1B). However, the purpose of this study was to identify a new chemotype that might act on IN via a novel mechanism, distinct from known active site inhibitors. Accordingly, we retrieved the chemical structure of the 130 hits and looked for a chemical pattern different from that of conventional strand transfer inhibitors. Interestingly, we found three stilbene disulfonic acids (NSC34931, NSC76027, and NSC638352) that inhibited 77.8%, 62.8%, and 67.2% of the IN ST activity at 10 µM, respectively (Figure 1A,B). Because NSC34931 was the most active and had previously been found to be antiviral in an HIV-1 cytopathic assay (http://dtp.nci.nih.gov, [7]), this compound was prioritized for further structure–activity relationship studies.

### 2.2. Biochemical Characterization of NSC34931 and Derivatives

Looking for related compounds within the Open Chemical Repository Collection, we selected NSC34931 (the initial hit), NSC34933, NSC47745, NSC163175, and NSC163 to perform a structure–activity relationship study (Table 1). First, we confirmed that NSC34931 was indeed a specific inhibitor, using a different assay to monitor HIV-1 IN activities. In the classic gel-based assay, NSC34931 was able to inhibit both the 3′-P and the subsequent coupled ST, having IC_50_ values of 320 nM and 180 nM, respectively (Table 1). NSC34931 was equally active in the presence of Mg^2+^ or Mn^2+^ as a metal cofactor (data not shown). Finally, using a precleaved substrate (bypassing the 3′-P step) did not impact the ST inhibition of NSC34931, which had an IC_50_ value of 230 nM ± 39 nM (data not shown). As a comparison, MA-DKA inhibited ST more efficiently when manganese was used instead of magnesium (IC_50_ values of 70 nM and 330 nM, respectively), and its IC_50_ value for the inhibition of 3′-P was 70 µM, regardless of what metal cofactor was used [4]. Thus, unlike the diketo acid family of inhibitors that selectively inhibit ST over 3′-P, stilbene disulfonic acid efficiently inhibited both 3′-P and ST.

Originally known as Cancer Chemotherapy National Service Center number, the NSC number (NSC #) is an identifying number assigned by the DTP.

Interestingly, removing the naphthalene groups was highly detrimental, and NSC163 and NSC163175 were inactive up to the highest tested concentration of 111 µM. Replacing this naphthalene with an ethoxybenzene was less drastic, having a 10-fold loss of potency, as NSC47745 exhibited an IC_50_ value of about 3 µM for both 3′-P and ST. Finally, NSC34933 represented only subtle rearrangements in the naphthalene substitutions, and it exhibited similar low to sub-micromolar inhibition of IN.

NSC34931 and NSC34933 were tested for their ability to block viral infection. NSC34931 was antiviral, having an EC_50_ value in the low micromolar range (3.07 µM), and exhibited only limited cytotoxicity, having a CC_50_ value of 59 µM (Table 1). This is in agreement with previous publications, including the AIDS screening data from the Developmental Therapeutics Program [7]. Although NSC34933 was slightly less potent than NSC34931 at inhibiting IN in vitro, it inhibited viral replication at sub-micromolar concentration, having an EC_50_ value of 0.6 µM. Interestingly, NSC34933 was also less toxic than NSC34931, having a CC_50_ above the highest concentration tested of 100 µM. As a result, the selectivity index of NSC34933 was >166 (Table 1). Altogether, these results demonstrate that stilbene disulfonic acid derivatives are sub-micromolar antiviral drugs that possess good selectivity indexes. In addition, we identified a new derivative, NSC34933, that has better cellular properties than the initially reported antiviral molecule NSC34931.

### 2.3. Stilbene Disulfonic Acid Derivatives Overcome Resistance to IN Strand Transfer Inhibitors

**Figure 2:**
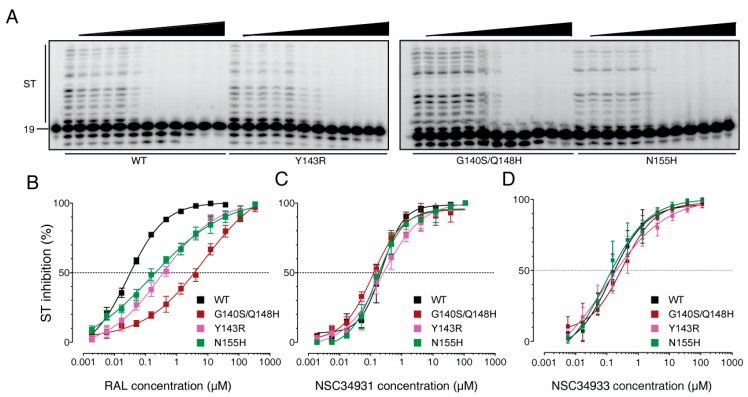
Activity of stilbene disulfonic acid derivatives against the ST activity of clinical RAL-resistant IN mutants. (**A**) Representative PAGE images showing NSC34931 inhibition of ST in the context of WT, Y143R, G140S/Q148H, and N155H RAL-resistant HIV-1 mutant integrases. (**B**–**D**) Quantitative analysis of the inhibition of ST by RAL, NSC34931, and NSC34933 derived from the densitometric analysis of gel-based experiments (including gels presented in panel A). Fitting curves and error bars represent the mean±SD from 10, 6, and 7 independent experiments for RAL, NSC34931, and NSC34933, respectively.

Next, we evaluated NSC34931 and NSC34933 against a panel of clinically relevant mutants resistant to IN active site inhibitors. We expressed the IN mutants G140S/Q148H, Y143R, and N155H, which correspond to the three major pathways responsible for RAL resistance in clinics [8,9]. When tested against these three mutant enzymes, RAL exhibited one to two orders of magnitude loss of potency compared to the WT enzyme, as expected (Figure 2B). Interestingly, NSC34931 inhibited the WT and all of the mutant enzymes at similar concentrations (Figure 2A). In detail, the IC_50_ values were 230, 120, 240, and 160 nM for the WT, G140S/Q148H, Y143R, and N155H mutants, respectively (Figure 2C). Similar results were obtained for NSC34933 (Figure 2D). These mutants remained completely susceptible to NSC34933, and they had IC_50_ values of 140, 320, 95, and 91 nM for the WT, G140S/Q148H, Y143R, and N155H mutants, respectively. These results confirm that stilbene disulfonic acid derivatives do not inhibit HIV-1 IN via the same mechanism as conventional strand transfer inhibitors, and the results suggest that such compounds could offer a therapeutic alternative to current IN-targeting drugs.

### 2.4. Molecular Mechanism of Action of Stilbene Disulfonic Acid Derivatives

**Figure 3:**
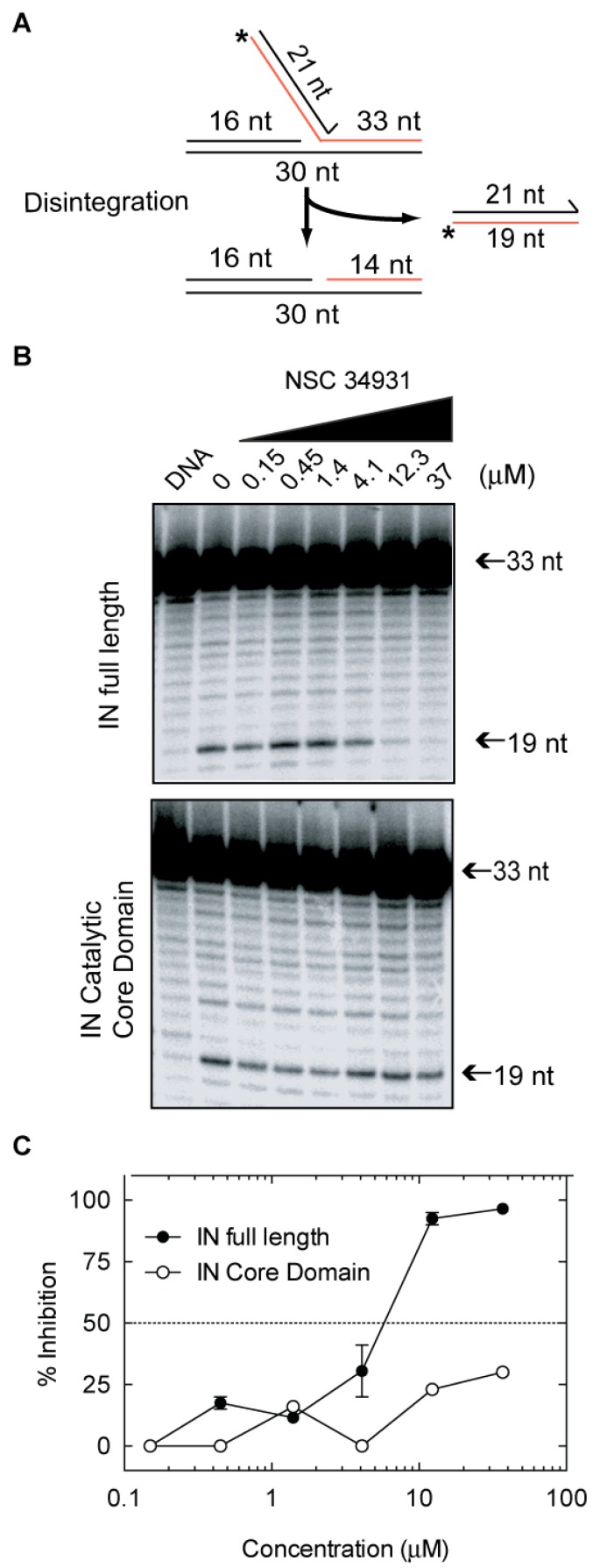
Inhibition of the disintegration reaction by NSC34931. (**A**) Schematic representation of the disintegration reaction. The length of each oligonucleotide is indicated (nt = nucleotide) (**B**) Representative PAGE image of the inhibition of disintegration by NSC34931 on full-length IN (top) or the isolated catalytic core domain (bottom). (**C**) Densitometric analysis of the gel pictures presented in B. Data represent mean ± SD from at least three independent experiments.

Conventional IN strand transfer inhibitors bind at the interface created by the protein, viral DNA, and two magnesium cations in the catalytic site of the enzyme [10]. Because stilbene disulfonic acid derivatives inhibit both 3′-P and ST with a similar efficiency, they may act outside the IN catalytic site. To determine if IN domains other than the catalytic core domain (CCD) are involved in the inhibitory activity of NSC34931, we tested this compound on disintegration, the only IN-mediated reaction that can be catalyzed by the isolated CCD. Disintegration is equivalent to a reverse-ST reaction (Figure 3A) [11]. Using a branched substrate, the WT enzyme as well as the isolated CCD catalyzed disintegration with similar efficiency (Figure 3B). NSC34931 inhibited disintegration catalyzed by the full-length IN at concentrations above 4.1 µM (Figure 3B,C). However, NSC34931 was unable to inhibit disintegration catalyzed by the CCD at concentrations up to 37 µM (Figure 3B,C). These experiments indicate that the *N*-terminal domain (NTD) and/or the *C*-terminal domain (CTD) are essential for IN inhibition by NSC34931.

**Figure 4:**
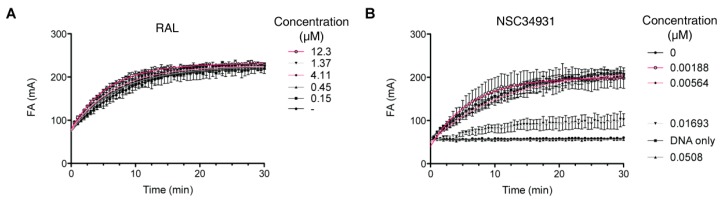
Measurement of IN-DNA binding over time using fluorescence anisotropy. Effect of RAL (**A**) and NSC34931 (**B**) on the DNA-binding property of IN. A three-fold serial dilution of RAL from 12.3 to 152 nM and from 50.8 to 1.88 nM for NSC34931 was used. In addition, controls with DMSO (0 µM) or without IN (DNA only) were included. Data represent the mean of three independent experiments.

Although the CCD alone harbors all of the structural determinants of the active site to support the catalytic activity, the NTD and CTD have been implicated in both the quaternary architecture of the enzyme and DNA binding. Because outer domains (NTD and/or CTD) appeared important in the inhibition of IN by NSC34931, we wondered if stilbene disulfonic acid derivatives might compete with DNA binding. To test whether NSC34931 competed with substrate binding, we developed a plate-based assay with a fluorescent DNA substrate. Over time, IN binding to its substrate slowed the rotation of the fluorophore compared to the free DNA, inducing an increase in anisotropy (Figure 4). As expected, RAL did not inhibit substrate binding, and increasing the concentration of the molecule even induced a slightly faster binding (Figure 4A). On the other hand, NSC34931 strongly and durably prevented IN-DNA binding, and full inhibition could be reached at concentrations as low as 51 nM (Figure 4B). Thus, inhibition of IN-DNA binding occurred at concentrations about 10 times lower than that necessary for the inhibition of IN catalytic activities, 3′-P, and ST.

**Figure 5:**
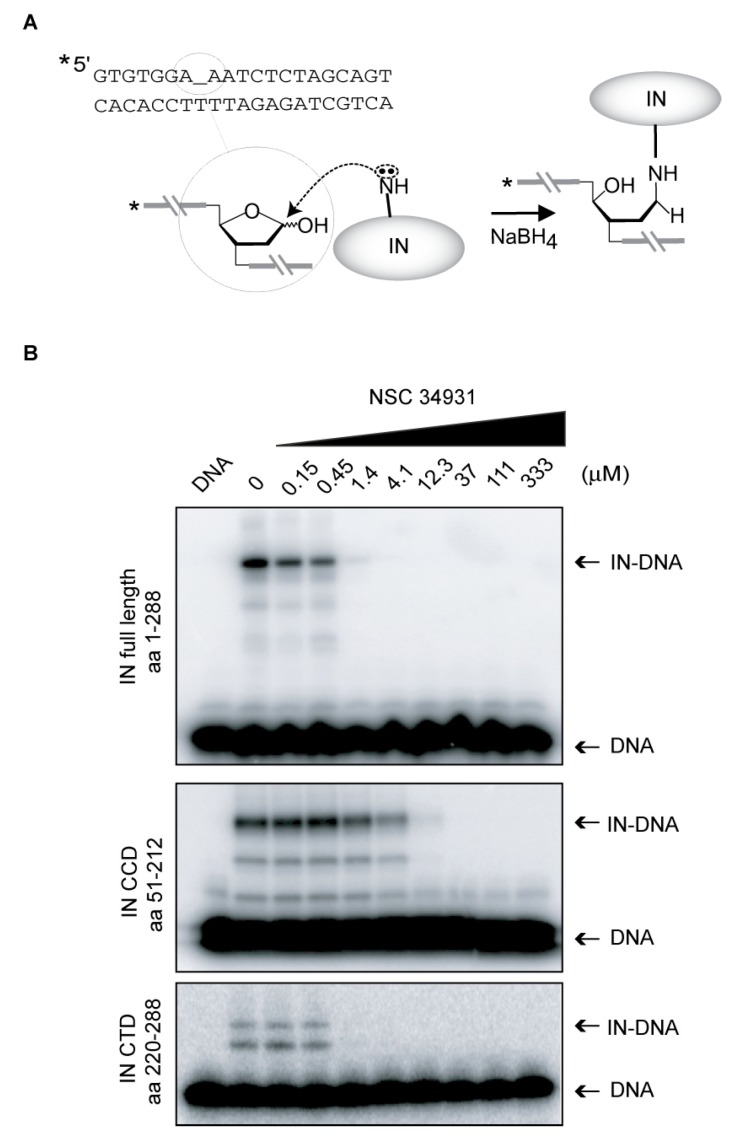
Inhibition of IN-DNA binding by NSC34931. (**A**) Principle of the Schiff base crosslinking assay. An abasic site was introduced by uracil DNA glycosylase in the DNA substrate at the -12 position. An IN nitrogen nucleophile (probably lysine) attacks the C1′-carbon of the abasic site. Rearrangement of the initial enzyme–DNA complex leads to the formation of a Schiff base intermediate that can be stabilized by reduction via NaBH_4_. The asterisk indicates the 5′-[^32^P]-label. (**B**) Representative SDS-PAGE image showing the inhibition by NSC34931 of crosslinking between IN and DNA using full-length IN (1–288), the isolated catalytic core domain (CCD) (51–212), or the isolated *C*-terminal domain (CTD) (220–288).

To gain further evidence that NSC34931 interferes with DNA binding, and to determine the role of outer domains in this inhibition, we evaluated the ability of NSC34931 to inhibit the formation of IN-DNA crosslinks using our previously described Schiff base assay [12,13,14]. Briefly, a deoxyuracyl nucleotide is incorporated in the DNA substrate to generate an abasic site via uracyl DNA glycosylase. IN forms a covalent complex (Schiff base), which can be stabilized by reduction with sodium borohydride (Figure 5A). Full-length IN, but also the isolated CCD or CTD domains, can crosslink DNA (Figure 4B). Consistent with anisotropy data, NSC34931 suppresses the formation of IN-DNA crosslinks in the context of the full-length IN (Figure 5B, upper gel). Of note, the effective dose to inhibit this reaction is similar to that needed to inhibit 3′-P or ST (about 0.45 µM). In contrast, the IN CCD requires a 10 times higher concentration of compound to prevent DNA crosslinking, confirming that the NTD or the CTD are important for NSC34931 inhibition. Lastly, the same experiment was conducted with the isolated CTD (Figure 5B, lower gel). Although the overall crosslinking efficiency was lower with the CTD compared to the full-length IN and the isolated CCD, NSC34931 inhibited the appearance of CTD-DNA crosslinks in the same concentration range than in the context of the full-length enzyme. Altogether, these results suggest that NSC34931 inhibits IN-DNA binding, and the CTD of IN plays a role in this competition with the substrate.

## 3. Discussion

Stilbene disulfonic acid derivatives have been described as nontoxic and noncarcinogenic [15]. They are used in pharmacology as antifungals [16], diuretics [17], neuroprotective agents [18], and anion channel blockers [19,20]. In the present study, we report a series of antiviral stilbene disulfonic acid derivatives that inhibited HIV-1 IN, by competing with viral DNA binding. The structure–activity relationship for the stilbene disulfonic acid derivatives revealed that their antiviral and anti-IN activities depend on the presence of symmetric aromatic moieties flanking both sides of the stilbene disulfonate core (Table 1). In this study, we have focused on NSC34931, because it is the most potent anti-IN compound among the series of analogs analyzed in vitro.

Inhibition of IN by NSC34931 exhibits some remarkable characteristics that set NSC34931 apart from current IN active site inhibitors. First, NSC34931 inhibits both 3′-P and ST with similar efficiencies, which is in contrast with the high ST-selectivity of active site inhibitors [21]. Second, NSC34931 inhibits RAL-resistant IN mutants G140S/Q148H, N155H, and Y143R, which are involved in the three major clinical resistance pathways to RAL (for review see [3]). The latter characteristic indicates that stilbene disulfonic acids have the potential to overcome clinical resistance to current and future active site inhibitors. Together with the DNA-binding inhibition, those differences are consistent with our finding that NSC34931 targets the IN out of its active site and probably involving the CTD. Targeting IN outside of its catalytic site may also be the only option to efficiently alleviate the appearance of clinical resistance to IN strand transfer inhibitors, similar to what was achieved with nucleoside and non-nucleoside reverse-transcriptase inhibitors.

Because the 3D structure of HIV-1 IN remains elusive, it is not possible to rationally design surface ligands that would impair IN functions. Still, LEDGF was the first and most described IN-interacting cellular cofactor [22]. It tethers the integration complex to the integration site, directing the selectivity of IN for highly transcribed regions of the genome. Additionally, because the IN-binding domain of LEDGF is targeting a dimer interface of IN, LEDGF stabilizes higher order oligomers, leading to IN activation in cells. Accordingly, molecules targeting the IN-LEDGF interface have been developed. These inhibitors have a complex mechanism of action, and they have been shown to be very useful tools to understand the role of IN during virus assembly and morphogenesis [22,23]. Because stilbene disulfonic acid derivatives affect DNA-binding, they should exhibit a simpler mechanism of action without affecting integration site selection (LEDGF tethering) or viral morphogenesis. From a more pharmacological point of view, inhibitors of the IN-LEDGF interaction have been shown to exhibit an additive effect with current active site inhibitors [24]. It is worth noting that when NSC34931 and NSC34933 were tested for synergy with an active site inhibitor (MK-2048), we could not see any cooperative effect, and the IC_50_ of MK-2048 was not affected in the presence of a subinhibitory dose of stilbene disulfonic acid derivatives (data not shown).

Stilbene disulfonic acids exhibited antiviral activity with good therapeutic indexes (e.g., >166 for NSC34933, Table 1). Diisothiocyanate of stilbene disulfonic acid (DIDS) has previously been reported to block HIV by inhibiting the CD4-gp120 interaction [25]. Nonetheless, it has been suggested that the antiviral naphtyldisulfonic acid dendrimer BRI2923 interferes with RT and/or IN based on time-of-addition experiments [26]. However, sulfonate, but not carboxylate, analogs could permeate cells. Thus, cellular targets responsible for the antiviral activity of NSC34931 and NSC34933 remain to be elucidated. Altogether, the low toxicity of stilbene disulfonic acid derivatives associated with both IN and viral entry inhibition could make these compounds prime candidates in the development of microbicides for topical application.

## 4. Materials and Methods

### 4.1. Chemicals 

Drugs were obtained from the National Cancer Institute Developmental Therapeutics Program (DTP-NCI, NIH). RAL and MK-2048 were purchased from Selleck Chemicals LLC (Houston, TX USA). Control inhibitor 118-D-24 was obtained through the NIH AIDS reagent program. All compounds were dissolved in 100% DMSO. Stock solutions (10 mM) were stored at −20 °C.

### 4.2. Oligonucleotides 

Oligonucleotides were purchased from Integrated DNA Technologies, Inc. (Coralville, IA, USA), purified on polyacrylamide gel through electro-elution and dissolved in water. The oligonucleotides 21T (GTGTGGAAAATCTCTAGCAGT) and 21B (ACTGCTAGAGATTTTCCACAC) correspond to the cleaved and noncleaved strands specifically recognized by HIV-1 IN.

Depending on the experiment, radiolabeling at the 5′-end was performed using T4 polynucleotide kinase (New England Biolabs, Ipswich, MA, USA) with [γ-32P] ATP (Perkin-Elmer Life and Analytical Sciences, Boston, MA, USA), according to the manufacturers’ instructions. Unincorporated isotopes were removed using mini quick spin oligo columns (Roche Diagnostics, Indianapolis, IN, USA). DNA duplexes were annealed using an equimolar ratio of the complementary strand 21B, heating to 95 °C, and slow cooling to room temperature.

### 4.3. Integrase Enzymes 

Recombinant enzymes were expressed in *E. coli* RosettaII (IPTG induction) and purified on nickel chelating column as described in [21]. Integrity and purity of wild type (WT) and mutant enzymes were checked by direct coomassie coloration of elution fractions on SDS-PAGE. Protein concentration was determined using a NanoDrop 2000 with the following parameters: molecular weight: 34kDa, extinction coefficient ϵ = 50,460 mol^−1^ cm^−1^ L.

### 4.4. Electrochemiluminescent Integrase Strand Transfer Assay 

This electrochemiluminescent plate-based assay was performed using a BioVeris M-SERIES Analyzer (Gaithersburg, MD, USA). DNA substrates were obtained from BioVeris and used according to the manufacturer’s recommendations. Briefly, a biotinylated donor DNA was incubated for 30 min at 37 °C in the presence of 250 nM of recombinant HIV-1 integrase. Complexes were bound to paramagnetic streptavidin-coated beads (M-280 Dynabeads). After addition of the drug, the integration reaction was initiated by addition of a ruthenium-labeled target DNA. The reaction was carried out for 60 min at 37 °C, before reading on the BioVeris M-SERIES Analyzer.

### 4.5. Integrase Reactions 

IN reactions were carried out by adding drugs or an equivalent volume of 100% DMSO (dimethyl sulfoxide, used as the drug solvent) to a mixture of 20 nM duplex DNA (21T/21B) and 400 nM IN in 50 mM MOPS pH 7.2, 7.5 mM MgCl_2_, and 14 mM 2-mercaptoethanol. Reactions were performed at 37 °C for 2 h and quenched by addition of an equal volume of loading buffer [formamide containing 1% SDS (sodium dodecyl sulfate), 0.25% bromophenol blue, and xylene cyanol]. Reaction products were separated in 16% polyacrylamide denaturing sequencing gels. Dried gels were visualized using a Typhoon 8600 (GE Healthcare, Piscataway, NJ, USA). Densitometric analyses were performed using the ImageQuant 5.1 software from GE Healthcare. Data analyses (linear regression, IC_50_ determination, and standard deviation) were performed using Prism 6.05 software from GraphPad.

### 4.6. Shiff Base Cross-Linking Assay 

Oligonucleotides 21T-12U containing a single uracil at the −12 positions were 5′γ-^32^P-, labeled as described above. After annealing with 21B, uracil DNA glycosylase was added to create an abasic site at the uracil position. The Schiff base crosslinking experiments were performed as described previously [12]. Inhibitors were preincubated for 20 min at room temperature with 400 nM WT IN, 7.5 mM MgCl_2_, 14 mM 2-mercaptoethanol, and 20 mM MOPS, pH 7.2. Abasic-site containing duplex DNA (final concentration, 20 nM) was added to each reaction and incubated at room temperature for 5 min. The crosslinks were reduced by adding 100 mM of freshly dissolved sodium borohydride (final concentration) before the addition of tricine-SDS gel loading buffer (1X final concentration). The crosslinked integrase–DNA products were loaded on 16% tricine SDS-PAGE gels (Invitrogen, Carlsbad, CA, USA). After migration of the samples, gels were treated similarly to sequencing gels (see above).

### 4.7. DNA-Binding Experiments 

DNA binding was measured using a plate-based assay as previously described [27]. The fluorescent probe used in this assay was obtained by annealing 21B to a specific 21T oligonucleotide, containing an AlexaFluor 488 modification at the 5′-end. Compounds or DMSO were incubated at room temperature for 5 min in the IN-activity buffer, in the absence or the presence of IN (400 nM). After addition of the DNA (10 nM), fluorescence anisotropy was measured every 30 s for 30 min using an Envision plate reader (Perkin Elmer, Waltham, MA, USA).

### 4.8. Antiviral Assays 

The HIV-1 replication assays were performed as described previously [28], using VSV pseudo-typed HIV-1 virus particles to infect MT4-LTR-EGFP cells that contain an enhanced green fluorescent protein (eGFP) gene under the control of the HIV-1 LTR promoter sequence. Successful HIV-1 infection results in viral Tat expression, which subsequently induces eGFP expression. Compounds inhibiting HIV-1 infection reduce EGFP expression as compared with the untreated HIV-infected control. A parallel cytotoxicity assay was performed on MT4-CMV-eGFP indicator cells containing an eGFP gene under the CMV early promoter. These cells constitutively express eGFP, and cytotoxicity is detected as decreased reporter gene expression.

## Figures and Tables

**Table 1:**
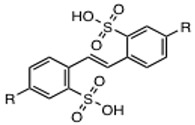
Summary of stilbene disulfonic acid derivatives activities. IN-catalyzed 3′-P and ST were monitored using a gel-based assay. IN-catalyzed 3′-P corresponds to the cleavage of the terminal dinucleotide at the 3′-end of the viral DNA mimic, while ST corresponds to the integration of the 3′-P product within another DNA molecule. Values derived from at least three independent experiments.

		In Vitro (µM, IC_50_ +/−SD)		Ex Vivo (µM)		
NSC #	R	3’-P	ST	Antiviral Activity	Cytotoxicity	Selectivity Index
				EC_50_	CC_50_	SI
34931	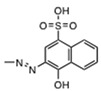	0.32 ± 0.12	0.18 ± 0.07	3.07	59.0	19.2
34933	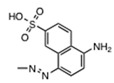	1.1 ± 0.3	0.5 ± 0.2	0.60	>100	>166
47745	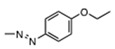	3.2 ± 1.5	3.1 ± 1.7	15.3	>100	>6.5
163175	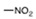	>111	>111	ND	ND	ND
163	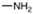	>111	>111	ND	ND	ND

Originally known as Cancer Chemotherapy National Service Center number, the NSC number (NSC #) is an identifying number assigned by the DTP.
